# Hypoxia reduces testosterone synthesis in mouse Leydig cells by inhibiting NRF1-activated StAR expression

**DOI:** 10.18632/oncotarget.14842

**Published:** 2017-01-27

**Authors:** Xueting Wang, Longlu Pan, Zhiran Zou, Dan Wang, Yapeng Lu, Zhangji Dong, Li Zhu

**Affiliations:** ^1^ Department of Biochemistry, Institute for Nautical Medicine, Nantong University, China; ^2^ Department of Rehabilitation of the Six People’s Hospital of Nantong, Jiangsu, China; ^3^ Co-Innovation Center of Neuroregeneration, Jiangsu Key Laboratory of Neuroregeneration, Nantong University, Nantong, China; ^4^ Key Laboratory of Neuroregeneration of Jiangsu and Ministry of Education, Co-Innovation Center of Neuroregeneration, Nantong University

**Keywords:** NRF1, StAR, testosterone synthesis, hypoxia, Leydig cells

## Abstract

Male fertility disorders play a key role in half of all infertility cases. Reduction in testosterone induced by hypoxia might cause diseases in reproductive system and other organs. Hypoxic exposure caused a significant decrease of NRF1. Software analysis reported that the promoter region of steroidogenic acute regulatory protein (StAR) contained NRF1 binding sites, indicating NRF1 promoted testicular steroidogenesis. The purpose of this study is to determine NRF1 is involved in testosterone synthesis; and under hypoxia, the decrease of testosterone synthesis is caused by lower expression of NRF1. We designed both *in vivo* and *in vitro* experiments. Under hypoxia, the expressions of NRF1 in Leydig cells and testosterone level were significantly decreased both *in vivo* and *in vitro*. Overexpression and interference NRF1 could induced StAR and testosterone increased and decreased respectively. ChIP results confirmed the binding of NRF1 to StAR promoter region. In conclusion, decline of NRF1 expression downregulated the level of StAR, which ultimately resulted in a reduction in testosterone synthesis.

## INTRODUCTION

In recent years, the growing number of male infertility aroused broad attention. Kamali et al. reported that out of 2492 infertile cases from 1993 to 2001, nearly 50.5% were related to male infertility, with only 11.6% were related to couple infertility [1]. Based on statistics released by the WHO, the prevalence of infertility is 10–15%, among which 35–40% of cases are related to male infertility disorders. Thus, it can be concluded that male fertility disorders play a key role in half of all infertility cases.

Among several physiological effects, studies suggested that hypoxia reduces fertility in humans. It was demonstrated that people exposed to 7821 m above the sea level presented sperm counts decrease and an increase in the number of dysmorphic spermatozoa [2]. Histological examination of rat testes after hypoxic exposure has shown the changes in testicular morphology and loss of spermatogenic cells [3, 4]. Until now the mechanism of hypoxia induced infertility was still unclear. One study performed by Madrid et al. in 2013 showed that high-altitude hypoxia induced plasmatic and testicular testosterone decreases since the 5th day [5]. As we know, testosterone plays an important role in the male reproductive system, by promoting sperm maturation and maintaining male secondary sex characteristics. Testosterone also promotes reproductive organ function, muscle protein synthesis, bone growth, calcium and phosphorus deposition and red blood cell formation. Studies have indicated that androgen deficiency led to not only male sexual dysfunction, erectile dysfunction, decreased reproductive capacity, but also cardiovascular disease [6], diabetes [7], osteoporosis and other diseases [8]. Thus, hypoxia-induced testosterone decrease might be one reason of male infertility.

Nuclear respiratory factor 1 (NRF1) is a transcription factor that regulates expression of a spectrum of genes required for mitochondrial respiratory function. It was reported that treatment with hypoxia mimetics CoCl_2_ or incubation with 2% O_2_ in 3T3-L1 adipocyte cells reduced NRF1 expression [9]. Interestingly, as a physiological dependence of mitochondrial gene, NRF1 mRNA level is low in many tissues but much higher in testis and lung [10]. Pilot experiments also showed a decrease trend of NRF1 expression in testis. We suppose that NRF1 decrease was a possible cause of the testosterone reduction.

Testicular Leydig cells are the main androgen-producing cells in mammals. 95% testosterone of a man is released by Leydig cells, which are found adjacent to the seminiferous tubules in the testis. In physiological condition, Leydig cell steroidogenesis is modulated by LH/hCG, which stimulates cAMP-PKA signaling pathway, leading to the transferation of cholesterol from the cytoplasm to the mitochondria. Then cholesterol is converted to pregnenolone and released into cytoplasm following by the synthesis of testosterone through 3β-hydroxysteroid dehydrogenase (3β-HSD) and 17β-hydroxysteroid dehydrogenase (17β-HSD) [11]. The transferation of cholesterol mediated by steroidogenic acute regulatory protein (StAR) is a rate-limiting step of testosterone synthesis, and StAR is the rate limiting protein of this process [12, 13]. Software analysis prompted the promoter region of StAR contained a NRF1 binding region, or an antioxidant response elements (ARE), indicating NRF1 promotes testicular steroidogenesis.

In this study, we describe that NRF1 is involved in testosterone synthesis; and under hypoxia, lower expression of NRF1 reduces StAR expression, leading to the decrease of testosterone synthesis. These findings provide a molecular mechanism for NRF1-mediated induction of testosterone production in testicular Leydig cells under hypoxia.

## RESULTS

### Distributions of NRF1 in different organs after hypoxia treatment

**Figure 1:**
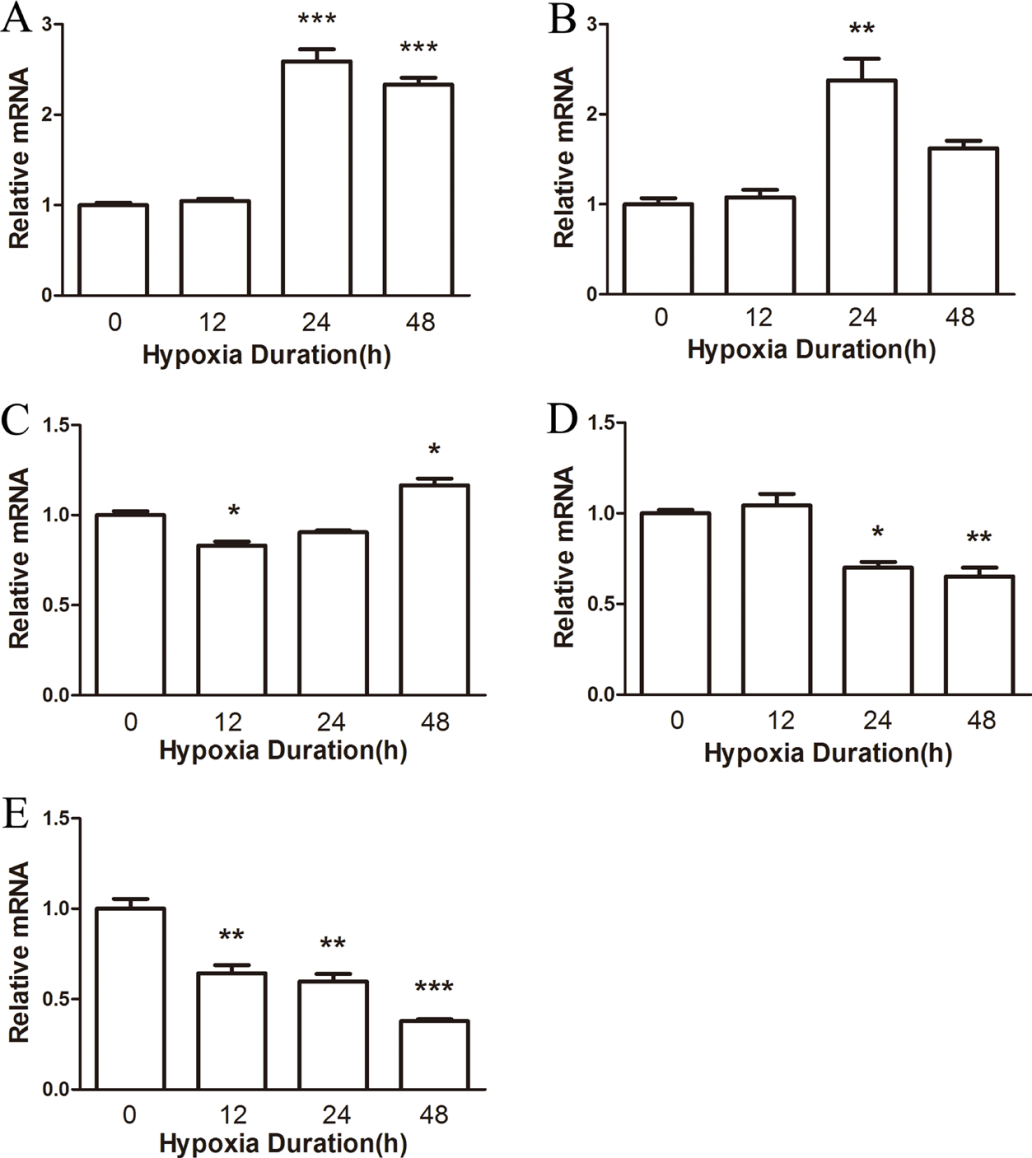
The mRNA level of NRF1 on different tissues after hypoxia treatment (8% O2) for 0, 12, 24 and 48 h The mRNA level of NRF1 in the heart (**A**), liver (**B**) and brain (**C**) increased after hypoxia treatment of male Balb/c mice. In the lung (**D**) and testis (**E**), NRF1 significantly decreased. *n* = 10, mean ± SD. *p < 0.05, **p < 0.01, ***p < 0.001.

**Figure 2:**
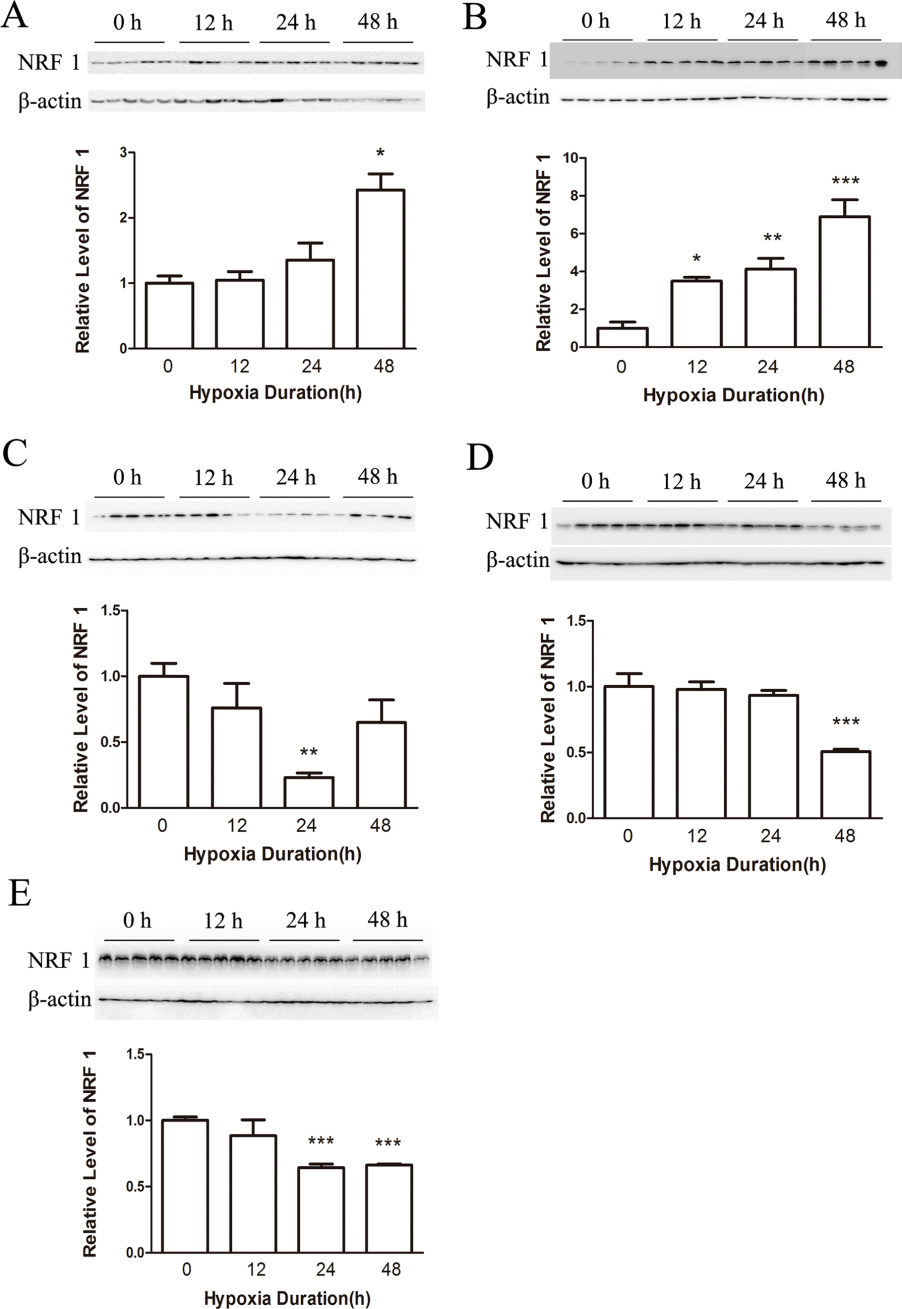
The protein level of NRF1 on different tissues after hypoxia treatment (8% O2) for 0, 12, 24 and 48 h The protein level of NRF1 in the heart (**A**) and liver (**B**) increased after hypoxia treatment of male Balb/c mice. In the brain (**C**), lung (**D**) and testis (**E**), NRF1 significantly decreased. *n* = 10, mean ± SD. *p < 0.05, **p < 0.01, ***p < 0.001.

To determine the effects of hypoxia on male mice, we first examined the distribution of NRF1 in different organs after hypoxia treatment. Organs including heart, liver, brain, lung and testis were collected to test the distributions of NRF1. We used Real-time PCR and Western Blot to detect the mRNA level and the protein level. As shown, the expression level of NRF1 in the heart (Figures 1A and 2A), liver (Figures 1B and 2B) and in the brain (Figure 1C) increased. On the contrast, the levels in the lung (Figures 1D and 2D) and the protein level in the brain (Figure 2C) significantly decreased. Interestingly, the decreased NRF1 level in testes were related with time (Figures 1E and 2E).

### Changes in NRF1 level and testosterone synthesis of Leydig cells under hypoxia condition

**Figure 3:**
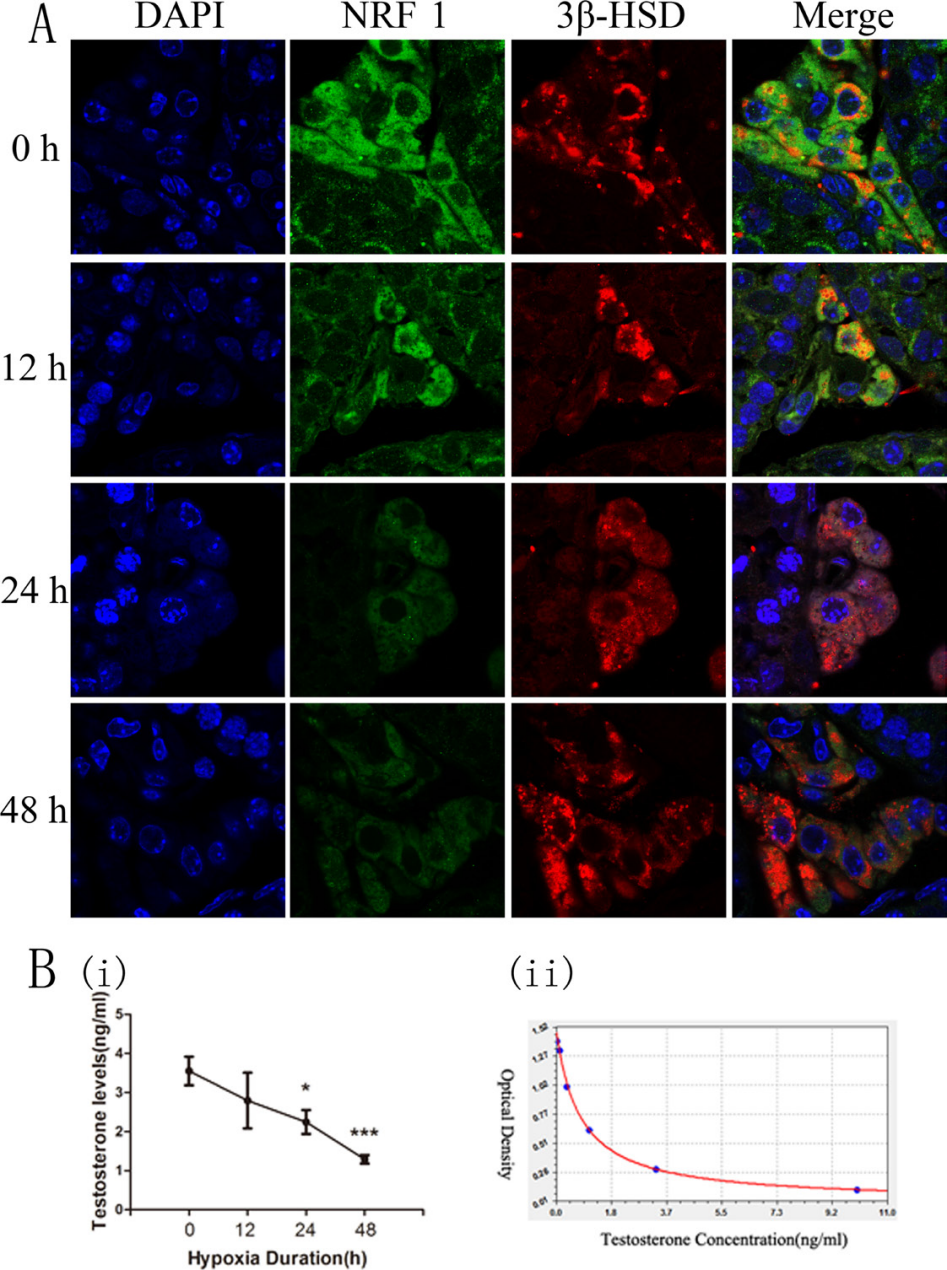
The expression of NRF1 in testis and the serum testosterone concentration of mice after hypoxia treatment (8% O_2_) for 0, 12, 24 and 48 h (**A**) NRF1 in Leydig cells in mice was expressed in cytoplasm and NRF1 significantly decreased after hypoxia treatment. Blue fluorescence represented cell nucleus of testicular sections stained by DAPI, red fluorescence indicated the location of the Leydig cells by 3β-HSD and green fluorescence represented NRF1 protein. (**B**) ELISA results showed that the serum testosterone levels were lower under hypoxia situation. *n* = 10, mean ± SD. *p < 0.05, *** *p* < 0.001 (i). ELISA Kit standard curve accords with Logistic curve, *r* = 0.99953991 (ii).

Testicular tissue is mainly composed of three cell types, Sertoli, Leydig, and spermatogenic cells. We measured the expression levels of NRF1 in different cell types by immunofluorescence technique. Positive 3β-HSD staining results confirmed Leydig cells, which also showed a much higher NRF1 level (Supplementary Figure 1). We were interested in the change of NRF1 level of Leydig cells after hypoxia treatments. So we also utilized the immunofluorescence technique for staining 3β-HSD (red) and NRF1 (green) of testicular tissue of mice treated with hypoxia. NRF1 was expressed in cytoplasm and NRF1 significantly decrease after hypoxia treatments (Figure 3A).

The main function of Leydig cells is the generation of testosterone. 95% of the testosterone is synthesized here. So the content of serum testosterone reflects the ability of Leydig cells to product testosterone. To measure the serum testosterone concentration, we collected mice serum and took the ELISA method. Results showed that the concentration of serum testosterone was decreased under hypoxia condition (Figure 3B).

**Figure 4:**
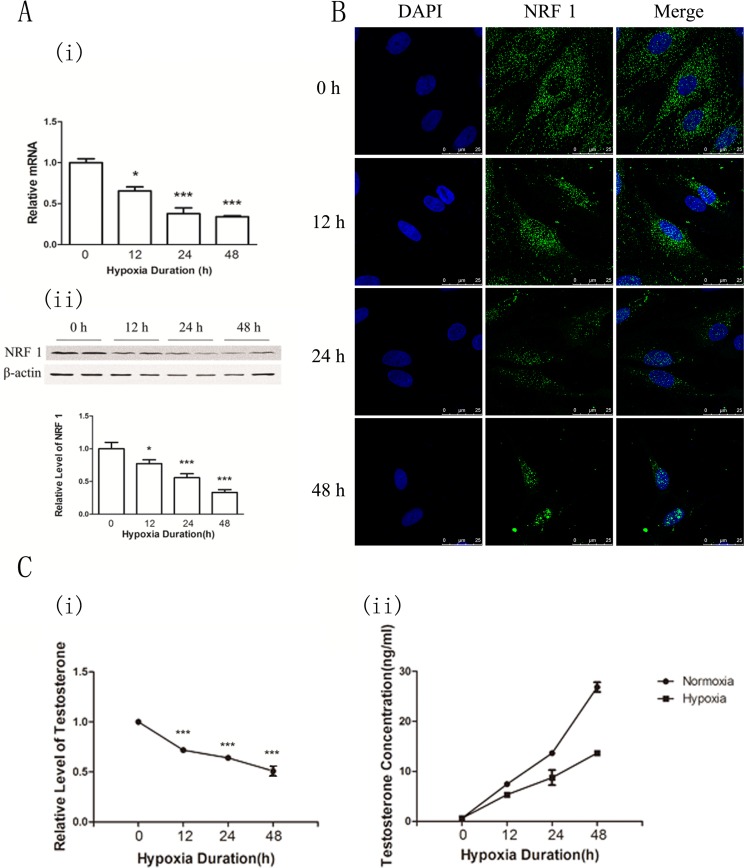
NRF1 levels and the testosterone concentration in the supernatant of primary cultured Leydig cells after hypoxia treatment (1% O_2_) for 0, 12, 24 and 48 h (**A**) The mRNA expressions of NRF1 were decreased after hypoxia treatments. *n* = 6, mean ± SD. *p < 0.05,**p < 0.01(i). The protein levels of NRF1 were decreased after hypoxia treatments. *n* = 6, mean ± SD. *p < 0.05,***p < 0.001(ii). (**B**) The expression of NRF1 on the Leydig cells was decreased after hypoxia treatment. Blue fluorescence represented nucleus of Leydig cells by DAPI and green fluorescence represented NRF1 protein. (**C**) Relative level of testosterone under hypoxia comparing to normoxia. ELISA results showed that the serum testosterone levels were lower under hypoxia situation *n* = 5, mean ± SD., ***p < 0.001 (i). The concentration of testosterone under hypoxia and normoxia condition. *n* = 5, mean ± SD. (ii). ELISA Kit standard curve accords with Logistic curve, *r* = 0.99953991 (iii).

To confirm the hypoxia effects of the NRF1 level and testosterone synthesis on Leydig cells, we isolated primary Leydig cells, which were treated with hypoxia (1% O_2_ concentration). The purity of the cultured Leydig interstitial cells (Leydig cells) was higher than 98% by immunofluorescence (Supplementary Figure 2). Leydig cells were kept under hypoxia condition for 0, 12, 24, 48 hours. NRF1 level was detected by real time-PCR, Western blot and ICC. Testosterone concentration was detected by Elisa. Results showed that the concentrations of serum testosterone were decreased under hypoxia condition and the NRF1 levels were decreased at both the mRNA and the protein level after hypoxia treatment (Figure 4A). Besides, the immunofluorescence results also suggested NRF1 showed obvious nuclear translocation after hypoxia treatment while the total expression decreased (Figure 4B). The testosterone of Leydig cells in the culture medium was detected by ELISA. The concentrations of testosterone were decreased under hypoxia condition (Figure 4C), which showed similar trend to the NRF1 changes. All results were consistent with the results of the *in vivo* experiments.

### NRF1 regulating the testosterone synthesis under normoxia and hypoxia conditions

**Figure 5:**
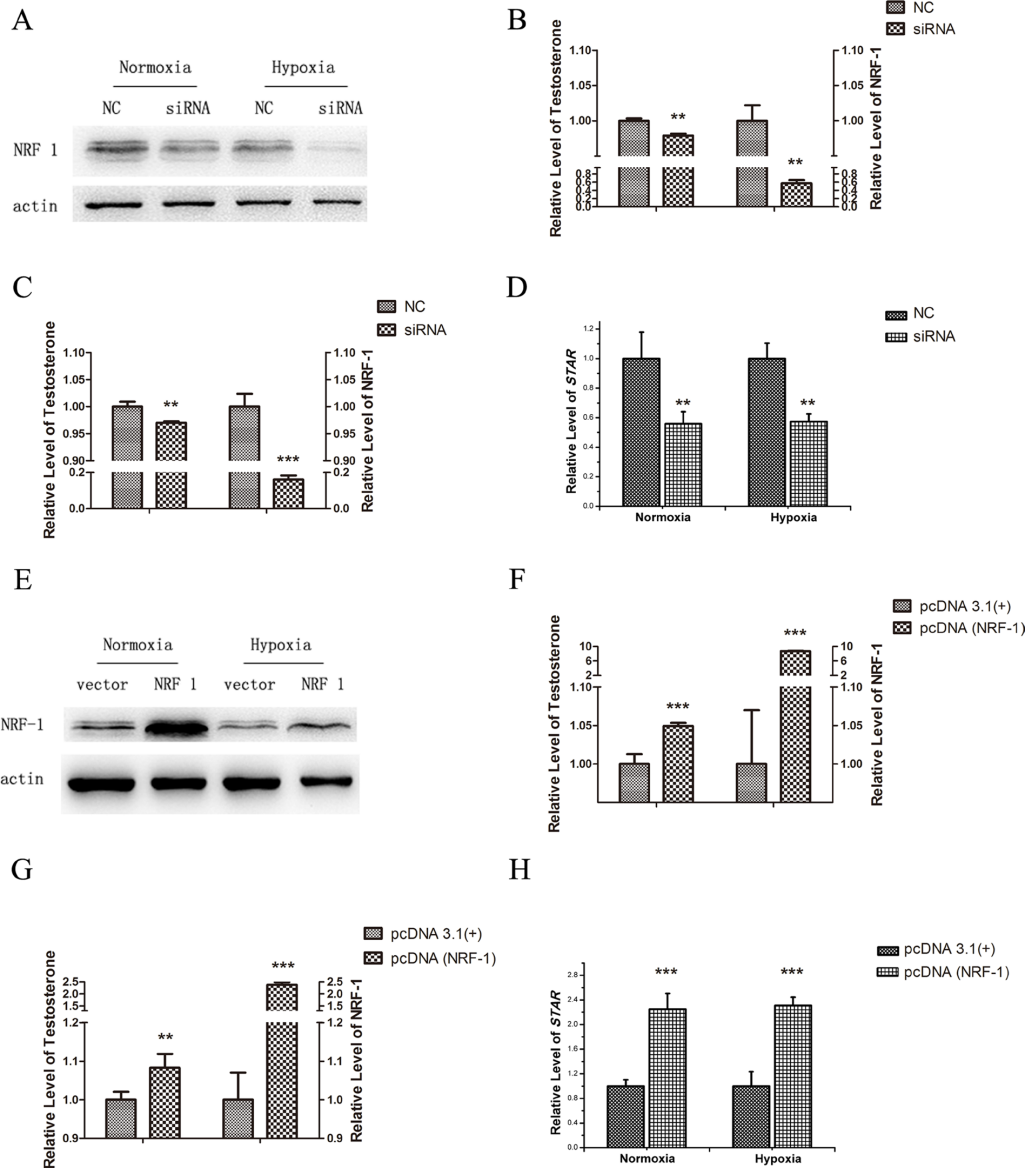
Testosterone release and StAR level of TM3 cells after the regulation of NRF1 under normoxia or hypoxia (1% O_2_, 24 h) NRF1 silencing in TM3 (**A**) markedly downregulated the release of testosterone under both normoxia (**B**) and hypoxia (**C**) environment, while the StAR expression followed the same trend (**D**). Overexpression of NRF1 (**E**) resulted in a higher testosterone release under both normoxia (**F**) and hypoxia (**G**) environment. Expressions of StAR showed the same trends (**H**). *n* = 5, mean ± SD., **p < 0.01, ***p < 0.001.

Both NRF1 and the testosterone were decreased in both testis and cultured Leydig cells. The trend of testosterone was consistent with the change of NRF1. Therefore, we hypothesized that NRF1 might be related to the synthesis of testosterone. Transfection with NRF1 small interfering RNAs and NRF1 overexpression plasmid was followed by normoxia or hypoxia treatment. The testosterone in the medium was detected by ELISA. As evident from Figure 5A, the siRNA-NRF1 brought about 40% decrease in NRF1 protein under normoxia and 85% decrease under hypoxia. Interestingly, siRNA-NRF1 transfected cells showed a significant decrease of testosterone levels under both normoxia and hypoxia as shown in Figure 5B and 5C. As evident from Figure 5E, 5F and 5G, overexpression of the NRF1 significantly increased the testosterone levels under both normoxia and hypoxia.

### NRF1 promoting StAR expression resulting in the relative changes of testosterone synthesis

Previous studies showed that NRF1 regulated gene transcription [14]. Therefore, we next determined whether NRF1 regulated the expression of genes involved in the pathway of testosterone synthesis. The potential binding sites of NRF1 to the regulatory region of StAR were found by Matlnspector software analysis. StAR is the rate-limiting step in the production of steroid hormones, which mediates cholesterol transfer into the mitochondria. As evidence from Figure 5D, StAR showed a significant decrease (50% under normoxia and 60% under hypoxia), similar to the NRF1 and testosterone trends. As illustrated in Figure 5H, StAR showed a significant increase (2.25 fold under normoxia and 2.3-fold under hypoxia), similar to the NRF1 level.

To identify the *in vivo* binding of NRF1 to StAR promoter, we used the CHIP analysis and amplified the sequence ranging from −450 to −20. Amplicons containing the already characterized NRF1 sites in the cyt c promoter were used as a positive control [15].

**Figure 6:**
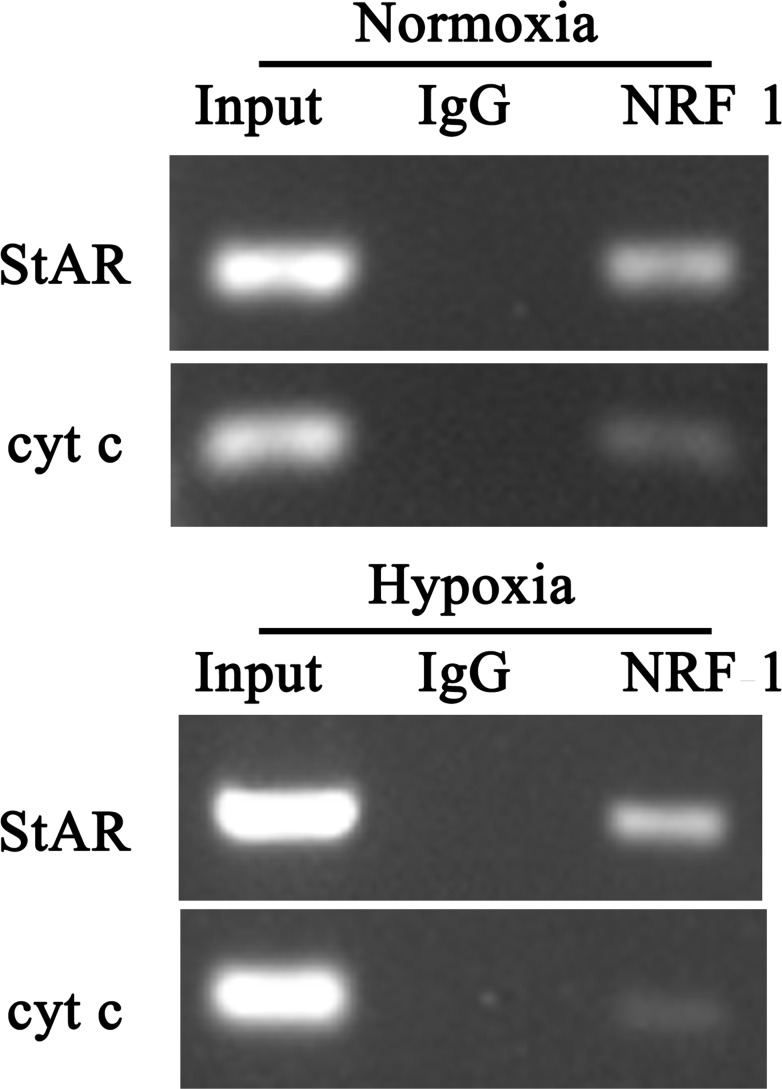
ChIP analysis for StAR promoter region Anti-NRF1 antibody was used for the ChIP assay and quantification of immunoprecipitated DNA fragments was performed by real-time PCR. The PCR product was obtained from the chromatin without immunoprecipitation reaction as group Input and IgG was as negative control. ChIP confirmation of occupancy of the NRF1 in StAR gene promoters using antibody against NRF1under normoxia or hypoxia (1% O_2_) for 24 hours.

As shown in Figure 6, immunoprecipitated StAR promoter fragments were obtained using specific NRF1 antibody in both normoxia and hypoxia groups, with respect to IgG antibody control samples. The specificity of the enrichment obtained for the analyzed promoter was confirmed by the presence of a significant signal depending on NRF1 antibodies using cyt c primers. The ChIP assay confirmed that NRF1 binding sites existed in the StAR promoter. The binding sequences of NRF1 to the StAR promoter was determined from −600 − +25.

**Figure 7:**
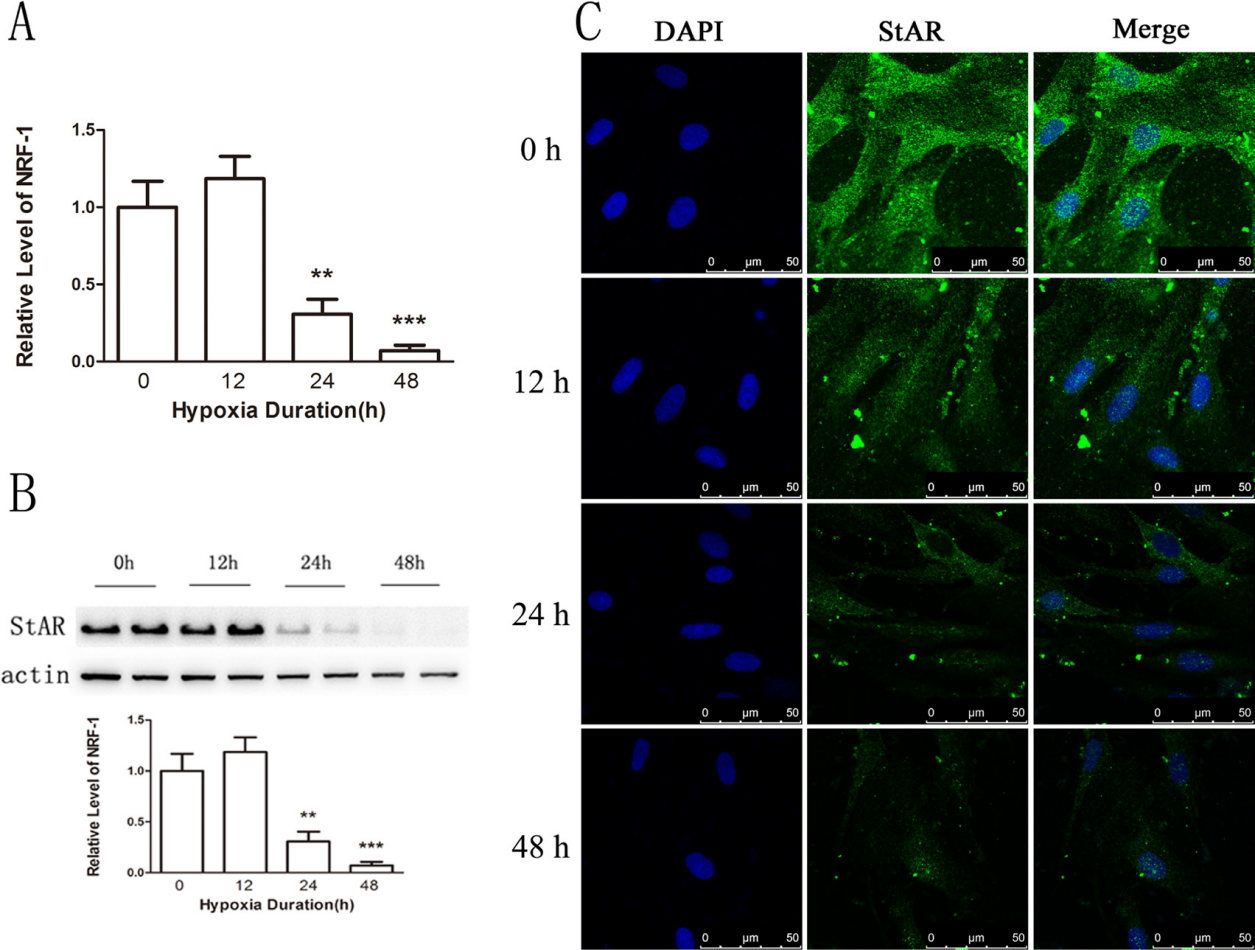
The expression of StAR on primary Leydig cells (**A**) The expression of StAR was decreased at the mRNA level. *n* = 6, mean ± SEM. **p < 0.01,***p < 0.001. (**B**) The expression was decreased on the protein level. *n* = 6, mean ± SEM. **p < 0.01,***p < 0.001. (**C**) The expression of StAR on the Leydig cells was decreased after hypoxia treatment. Blue color represented testicular sections counterstained by DAPI and green fluorescence represented StAR protein.

**Figure 8:**
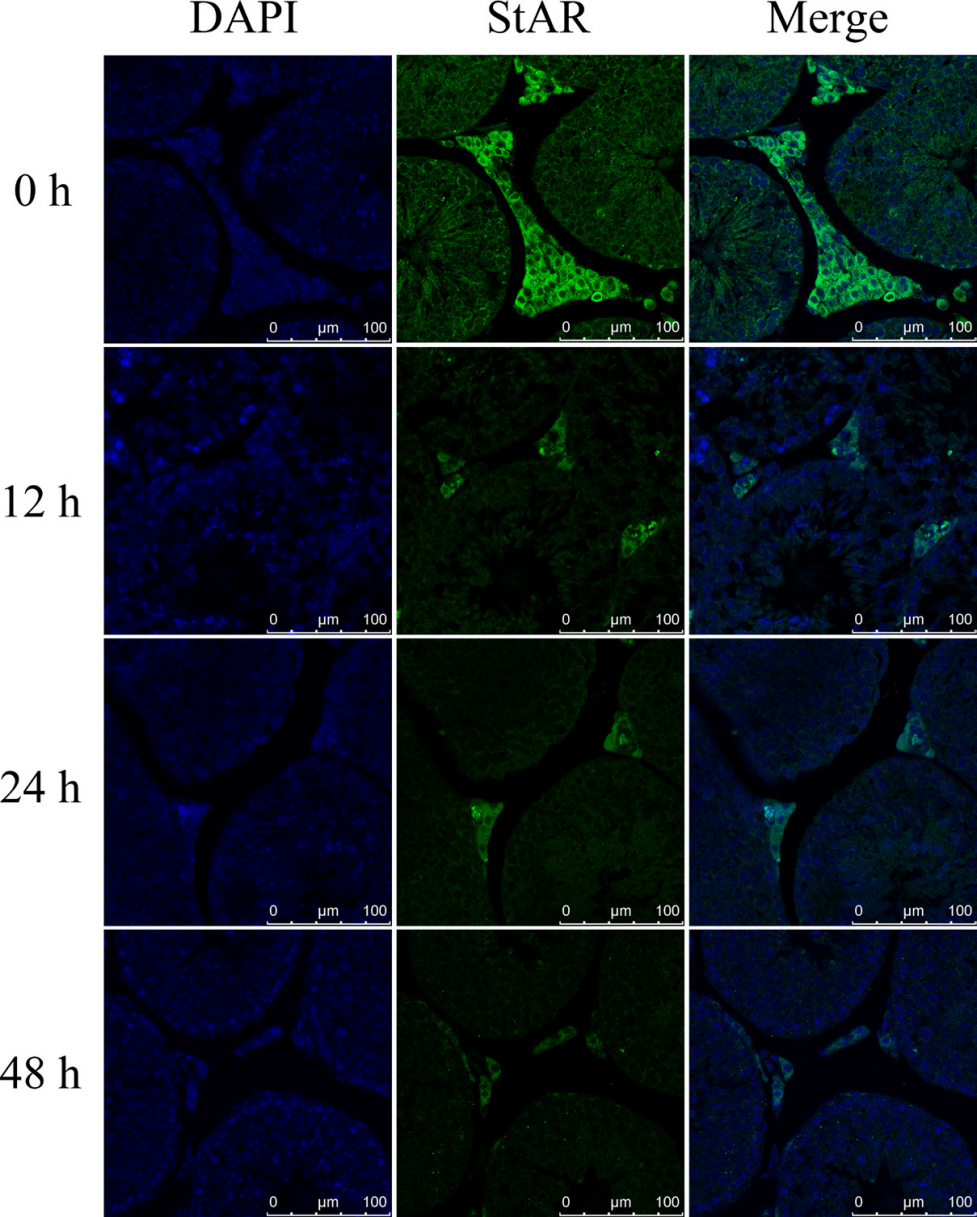
The expression of StAR in Leydig cells *in vivo* after hypoxia treatment Blue color represented testicular sections counterstained by DAPI and green fluorescence represented StAR protein.

To confirm that NRF1 decreased StAR gene transcription under hypoxia condition, we determined StAR expression *in vitro* and *in vivo* after hypoxia treatments. The primary Leydig cells were under hypoxia treatment for 0, 12, 24, 48 hours and StAR was detected. Results show that the expression of StAR was decreased at both the mRNA and the protein level after hypoxia treatment (Figure 7), which was consistent with the changes of NRF1 (Figure 4A and 4B). Besides, the immunofluorescence results also suggested StAR decreased in Leydig cells *in vivo* after hypoxia treatment (Figure 8).

## DISCUSSION

Snoring is the most common hypoxia status people experience when in sleep. Also, people working in offices tend to keep seated for long periods of time, which slows blood circulation to create hypoxia. Studies have shown that hypoxia affected respiratory, circulatory and endocrine systems [16, 17]. Hypoxia reduced the weight of thyroid [18], affected the function of hypothalamus-pituitary- adrenal axis [19], raised adrenocorticotropic hormone level [20]. Liao et al. reported high altitude hypoxia induced a significant reduction in the level of testosterone in male rats [21]. Hwang et al. found acute hypoxia upregulated testosterone release, on the contrary, chronic hypoxia downregulated testosterone release [22]. Madrid et al. in 2013 reported that high-altitude hypoxia induced plasmatic and testicular testosterone decreased from the 5th day [5]. Liu et al. reported in 2012 that in hypoxic rats, serum testosterone levels decreased and mRNA and protein expressions of testosterone biosynthesis related genes downregulated [23]. In this study, we determined the hypoxia effects on testosterone in Balb/c mouse serum and primary Leydig cells. Results showed a similar trend that hypoxia reduced the testosterone level, consistent with those previous reports.

Although early studies have indicated that hypoxia had a serious impact on the endocrine system, there are few reports about the mechanism of the testosterone reduction induced by hypoxia. Satoh et al. identified 2,470 protein-coding genes in SK-N-SH human neuroblastoma cells, in whose promoter regions existed the NRF1-binding consensus sequence. All those genes played a pivotal role in regulation of extra-mitochondrial biological processes, including splicing, cell cycle, RNA metabolism, protein translation initiation, DNA damage repair, and ubiquitin-mediated protein degradation [26]. Our previous studies firstly reported that NRF1 targeted HIF-1α and repressed hif-1α transcription [24], indicating NRF1 might be another important factor of hypoxia response. To evaluate the effect of NRF1 on testosterone synthesis, we overexpressed NRF1 with an overexpression plasmid and silencing NRF1 by transfection of NRF1 small interfering RNAs, and then detected testosterone concentration in supernatant of TM3 cells. No matter under normoxia or hypoxia condition, testosterone concentration was along with the change of NRF1 level (Figure 5). We tried to knock out NRF1 in HEK 293T cells using the CRISPR/Cas9 method to find cells with complete loss of NRF1 unable to survive (data not shown). There are no reports about NRF1 knock out mice, we suppose NRF1 knock out is lethal to both cells and animals.

In our previous study, we found testosterone released by TM3 cells increased under hypoxia, which was opposite to the results of *in vivo* and the primary Leydig cells. But the results consisted with the report of Hwang et al. in 2007. They found that TM3 cells synthesized testosterone in need of hCG stimulation, which increased vascular endothelial growth factor (VEGF) production under hypoxia condition, leading to the testosterone increase. This stimulatory effect was abolished by the administration of anti-VEGF antibody [25]. Minet et al. reported hCG stimulated ERK 1/2 phosphorylation, upregulating the testosterone level in TM3 cells [26]. Results indicated that TM3 cells were not an ideal model to study the hypoxia response of Leydig cells, since hCG “covered up” the actual effect of hypoxia.

Another interesting problem is how NRF1 affected testosterone synthesis. Bioinformatics analysis revealed that 5 testosterone synthesis related genes, whose promoter region had the potential binding site of NRF1, including Insulin-like 3, Nur 77, Peripheral-type benzodiazepine receptor (PBR), 3β-HSD and StAR. Primary experiments indicated that StAR was reduced most significantly under hypoxia, while other enzymes like 3β-HSD did not show such reduction. Insulin-like 3 is a peptide hormone in male mammals secreting by adult Leydig cells [27]. Insulin-like 3 was independent of control via gonadal hormone, functioning like an anti-apoptotic or survival factor in germ cells in the testis and ovary [28]. Nur77 has been shown to stimulate genes involved in steroidogenesis in Leydig cells, including StAR and 3β-HSD [29]. In Leydig cells, PBR co-act with StAR mediated cholesterol transfer into mitochondria [11]. The transferation of cholesterol is a rate-limiting step of testosterone synthesis, and StAR is the rate limiting protein of this process [12, 13], which is the protein of interest for its role in hypoxia-induced testosterone reduction. *In vivo* and *in vitro* experiments verified that StAR decreased in trends of NRF1 under hypoxia (Figures 7 and 8). ChIP analysis confirmed that NRF1 bond to StAR promoter region (Figure 6).

Some studies focused on the role of hypoxia in StAR. Liu et al reported in 2012 that in hypoxic rats, expression StAR and 3β-HSD were downregulated [23]. Su et al. reported that increases in plasma leptin inhibited the expression of StAR and steroidogenic enzymes and attenuate adrenal responsiveness in hypoxic fetuses [30]. Zhang et al. reported that an activated NADPH downregulated StAR expression in the hypoxia testis, inducing testicular damage [31]. Kowalewski et al. found inhibition of expression of HIF-1α suppressed StAR expression. HIF-1α appeared to be a positive regulator of StAR [32]. Our work found NRF1 promoted StAR expression under normoxia and hypoxia for the first time. The downregulation of StAR caused by lower NRF1 was the reason of, at least in part, testosterone reduction under hypoxia.

In Figure 1, it was shown that NRF1 in different tissues had different changes after hypoxia treatment. It might because of the complicate function of NRF1 in different tissues. The reasons of those different changes and the possible results caused by NRF1 were not revealed in this work. Considering the important role of NRF1 played in cells, its reduction should cause a lot of damages to Leydig cells. There are questions remaining to be answered, including what proteins are affected, how the mitochondria will respond, and what is the fate of Leydig cells along with the hypoxia duration.

## MATERIALS AND METHODS

### Animals and treatment

Male Balb/c mice (*n* = 60) weighing 20–25 g were supplied from Experimental Animal Center of Nantong University, China. All these mice were accommodated for one week before experiments with a 12 h:12 h light–dark cycle, and then were randomly divided into 4 groups with 15 mice per group: normoxia control group and 12, 24, 48 hypoxia condition group. Mice were confined in a sealed container in which 8% oxygen concentration were supplied for 0, 12, 24, 48 h. Then all animals were anaesthetized by 10% chloral hydrate with intraperitoneal injection. For the 10 mice in each group, blood samples were obtained from abdominal aorta, and samples of testis tissues were harvested for real-time PCR and Western Blot. Hearts of the other 5 mice in each group were instilled *in vivo* with 4% citromint. Testis paraffin section was made to observe the Immunofluorescence staining.

All the studies reported here was submitted to ethics committee on animal experimentation in Nantong University and all procedures were approved according to the Animal Care and Use Committee of Nantong University and the Jiangsu Province Animal Care Ethics Committee (Approval ID: SYXK(SU)2007-0021).

### Isolation and treatment of primary Leydig cells

The method used for the isolation and purification of Leydig cells was modified from one previously described for testis Leydig cells extraction [33]. Briefly, decapsulated Balb-c mice testes were immersed in 0.1% collagenase-PBS and were shaken for 30 min at 200 rpm, 37°C. After dissociation, the seminiferous tubules were removed by filtration through the wire on the mesh 200 to get cells suspension. The filtrate was centrifuged at 1200 rpm for 7 min at 4°C and cells were collected into medium of 27:3:20 (v/v/v) of Percoll: 9% NaCl saline solution: DMEM-F12 medium (Hyclone, GE Healthcare Life Sciences, Logan, Utah) and centrifuged at 20,000 × g for 60 min at 4°C. Cells were resuspended at 5 × 10^5^ cells/ml in DMEM-F12 medium containing 5% Newborn Calf Serum (Gibco, Grand Island, New York) for subsequent analysis. The collected cells were characterized with immunofluorescence stainning. More than 98% of the visualized cells were positive.

Finally, cells were added into 35 mm dishes containing 5 ml culture medium and maintained at 34°C in humidified atmosphere composed of 95% air, 5% CO_2_. After 12 h adherence, the dishes were divided into 4 groups receiving 1% O_2_, 5% CO_2_ incubation for 0, 12, 24, 48 h in an Invivo_4_ hypoxia chamber (Ruskinn Technologies, Leeds, UK). Cell supernatants were collected for testosterone concentration assay. Protein and mRNA were extracted for Western blot and real-time PCR.

### Culture and treatment of TM3 cells

TM3 cells (CRL-1714, ATCC, RRID:CVCL_4326) were a kind of non-tumorigenic cell line derived from mouse testis, which secret testosterone in response to Luteinizing hormone (LH) and human Chorionic gonadotropin (hCG). The mechanism is similar to primary Leydig cells. Cells were characterized with immunofluorescence staining. Cells were cultured in 1:1 mixture of Ham-12 and Dulbecco’s MEM (Hyclone, GE Healthcare Life Scinces, Logan, Utah), containing 5% horse serum (Gibco, Grand Island, New York), 2.5 % Fetal Bovine Serum (Hyclone, GE Healthcare Life Scinces, Logan, Utah), in 35 mm dishes. Cells were cultured at 37°C in humidified atmosphere composed of 95% air, 5% CO_2_. After 12 h adherence, the dishes were divided into 4 groups receiving 1% O_2_, 5% CO_2_ incubation for 0, 12, 24, 48 h in an Invivo_4_ hypoxia chamber (Ruskinn Technologies, Leeds, UK). Cell supernatants were collected for testosterone concentration assay. Protein and mRNA were extracted for Western blot and real-time PCR.

### Testosterone concentration assay

Blood samples were placed in 1.5 mL microcentrifuge tubes at room temperature for 2 h and centrifuged at 2000 rpm for 20 min to collect serum. Testosterone concentration of both serum and cell supernatants was examined by a Parametre Testosterone Assay kit (KGE010, R&D Systems, Minneapolis, Minnesota).

### Total RNA extraction and real-time PCR

Total RNA was isolated from hypothalamus using Column Animal RNAout (TianDZ, Beijing, China) according to the manufacturer’s instructions. Purified total RNA (500 ng) was then reverse-transcribed using HiScript 1st Strand cDNA Synthesis Kit (Vazyme, Nanjing, China).

The primer sequences used were shown in Supplementary Table 1. Briefly, amplification was subsequently carried out by mixing 1 μL of cDNA product with 10 μL of 2 × FastStARt Universal SYBR Green Master (Roche Diagnostics, Mannheim, Germany), 2 μL of the primer pair mix (2 mM sense and 2 mM antisense) and 7 μL of RNase-free water. Real-time PCR was performed in Lightcycler 96 (Roche Diagnostics, IN, USA).

### Total protein extraction and Western blot

Harvested mouse testes, primary Leydig cells or TM3 cells were homogenized in the protein extraction buffer for extracting proteins. The homogenates were kept on ice for 30 min and centrifuged at 12,000 g for 30 min. Supernatants were collected and protein concentrations were determined by the Bradford method. About 40 μg of the extracted protein from each sample were separated by SDS-PAGE and electrophoretically transferred onto a PVDF membrane. Membranes were blocked in 2.5% BSA + 2.5% milk in TBS for 2 h at 37°C. Blocked membranes were incubated with NRF1 polyclonal antibody (ab175932, Abcam, Hong Kong, China), beta-actin monoclonal antibody (ab8227, Abcam, USA) or StAR monoclonal antibody (8449, Cell Signaling Technology, Danvers, Massachusetts) in TBST containing 3% BSA at 4°C overnight. Next, the membranes were washed 6 times with PBST, 5 min each time, followed by incubation for 1.5 h at room temperature with a secondary antibody (Jackson ImmunoResearch, West Grove, Pennsylvania) at a concentration of 1: 10000 in TBST containing 3% BSA. The chemiluminescence reaction was performed using SuperSignal West Pico (Thermo Scientific, Rockford, IL).

### Immunofluorescence of testis paraffin sections

The fixed testes were embedded in paraffin, sectioned at 5 μm and dried at 37°C overnight. Tissue sections were immersed in fresh xylene for 5 min twice at room temperature. Sections were washed in 100% ethanol for 5 min. Next, sections were rehydrated by sequentially immersing the slides through graded ethanol washes (100%, 95%, 85%, 70%, 50%) for 3 min each. Then sections were washed in 0.85% NaCl for 5 min and in PBS for 5 min. Samples were fixed in 4% methanol-free formaldehyde in PBS for 15 min followed by washing twice with PBS. Sections were blocked in 5% BSA in PBS for 2 h at 37°C. To detect NRF1 level, blocked sections were incubated with NRF1 mAB (ab175932, Abcam, Hong Kong, China) and 3β-HSD mAB (sc-30820, Santa Cruz, USA), which was a marker of Leydig cells, in PBST containing 5% BSA at 4°C overnight followed by washing with PBST 6 times for 5 min each time. Sections were incubated with Alexa Fluor 488-conjugated donkey anti-rabbit IgG (R37118, Life technologies Eugene, Oregon) and Alexa Fluor 555-conjugated donkey anti-goat IgG (A-21432, Life technologies, Eugene, Oregon) for 2 h at 37°C and were protected from light followed by washing 6 times with PBST for 5 min each time. Finally, the samples were stained with DAPI (Life technologies, Eugene, Oregon) as nuclear counterstain. To detect StAR level, blocked sections were incubated with StAR mAb (8449, Cell Signaling Technology, Danvers, Massachusetts) in PBST containing 5% BSA at 4°C overnight followed by washing with PBST 6 times for 5 min each time. Sections were incubated with Alexa Fluor 488-conjugated donkey anti-rabbit IgG (R37118, Life technologies, Eugene, Oregon) for 2 h at 37°C and were protected from light followed by washing 6 times with PBST for 5 min each time. Finally, the samples were stained with DAPI (Life technologies, Eugene, Oregon) as nuclear counterstain. The samples were analyzed with a microscope (Leica, SP8) for the detection.

### Immunocytochemistry of primary Leydig cells and TM3 cells

Cells were crawled on the slide and incubated with 1% O2 for 0, 12, 24 and 48 h. Then slides were fixed in 4% methanol-free formaldehyde in PBS for 15 min followed by washing twice with PBS. Slides were blocked in 5% BSA in PBS for 2 h at 37°C. Blocked samples were incubated with StAR mAb (8449, Cell Signaling Technology, Danvers, Massachusetts) or NRF1 mAB (ab175932, Abcam, Hong Kong, China) in PBST containing 5% BSA at 4°C overnight followed by washing with PBST 6 times for 5 min each time. Samples were incubated with Alexa Fluor 488-conjugated donkey anti-rabbit IgG (1723091, Life technologies, Eugene, Oregon) for 2 h at 37°C and were protected from light followed by washing 6 times with PBST for 5 min each time. Finally, the samples were stained with DAPI (Life technologies, Eugene, Oregon) as nuclear counterstain. The samples were analyzed with a microscope (Leica, SP8) for the detection.

### Chromatin immunoprecipitation

Chromatin Immunoprecipitation (ChIP) was performed using the EZ-ChIP Kit (Millipore, Temecula, California, USA) according to the manufacturer’s instructions. Briefly, TM3 cells were washed and fixed in culture medium containing 1% formaldehyde at room temperature for 10 min. The cells were collected and lysed to release the nucleus. Nuclei were then isolated by centrifugation before being subjected to sonication. The sonicated lysate was then immunoprecipitated with 2 μg of NRF1 antibodies (ab34682, Abcam, Hong Kong, China), or a negative control IgG at 4°C for 4 h. The pulled-down chromatin was washed, reverse-cross linked and purified. To the detection of the target gene StAR and the positive control cyt c, PCR reactions were performed using 5% of precipitated chromatin. Primers targeting promoter sequences were shown in Supplementary Table 2.

### Over expression or gene silencing of NRF1

The expression plasmids for wildtype NRF1 were constructed according to a method described previously [34]. All constructs were verified by sequencing. TM3 cells were seeded into 6- or 24-well plates in antibiotic-free medium the day before transfection. Each well of cells was transiently transfected plasmids using X-tremeGENE HP DNA Transfection Reagent (Roche Diagnostics, IN, USA). Experiments for gene silencing were performed using small interfering NRF1 (siRNA-NRF1). Mouse NRF1 specific siRNA duplex were designed and synthesized by Dharmacon (Dharmacon, Lafayette, CO, USA). X-treme GENE siRNA transfection reagent (Roche Diagnostics, IN, USA) was used for transfection following the manufacturer’s protocol.

### Statistical analysis

Results are represented as means ± SD from three independent experiments. Statistical significance was determined by one-way ANOVA, followed by the post-hoc Tukey multiple comparison test.

## SUPPLEMENTARY MATERIALS FIGURES AND TABLES



## Abbreviations

NRF1: nuclear respiratory factor 1
StAR: steroidogenic acute regulatory protein
3β-HSD: 3β-hydroxysteroid dehydrogenase
17β-HSD: 17β-hydroxysteroid dehydrogenase
ChIP: chromatin immunoprecipitation
LH: Luteinizing hormone
hCG: Human Chorionic gonadotropin
VEGF: vascular endothelial growth factor
PBR: peripheral-type benzodiazepine receptor.
