# Supplementary material for: Hypoxia reduces testosterone synthesis in mouse Leydig cells by inhibiting NRF1-activated StAR expression
Source: Oncotarget. 2017 Jan 27;8(10):16401–13. doi: 10.18632/oncotarget.14842 (PMC5369971; doi:10.18632/oncotarget.14842)
Supplement: Supplementary file 1 [file oncotarget-08-16401-s001.pdf]

Hypoxia reduces testosterone synthesis in mouse Leydig cells by inhibiting NRF1 -activated StAR expression

Supplementary Materials

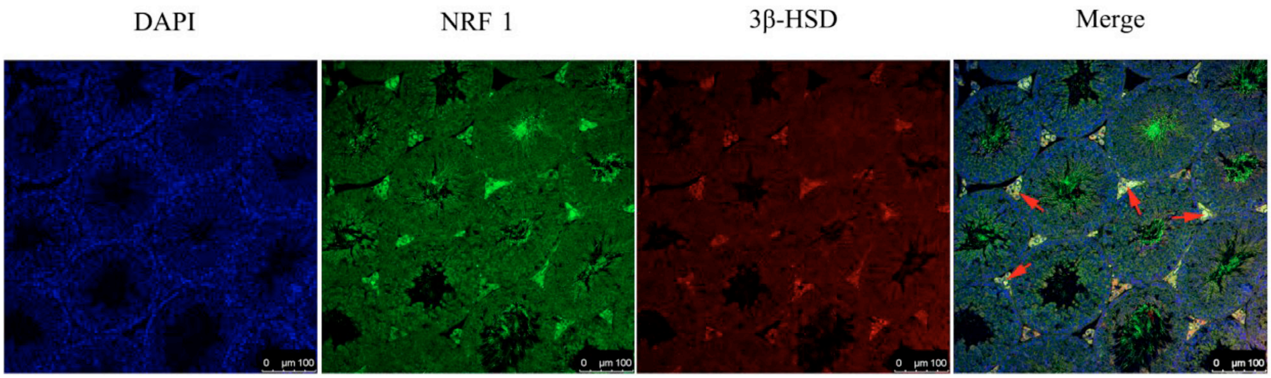

Supplementary Figure 1: The expression of NRF 1 in mouse testis. The expression of NRF 1 in Leydig cells was higher than those in spermatogenic cells and Sertoli cells. Blue fluorescence represented cell nucleus of testicular sections stained by DAPI, red fluorescence indicated the locations of the Leydig cells by 3β-HSD and green fluorescence represented NRF 1 protein.

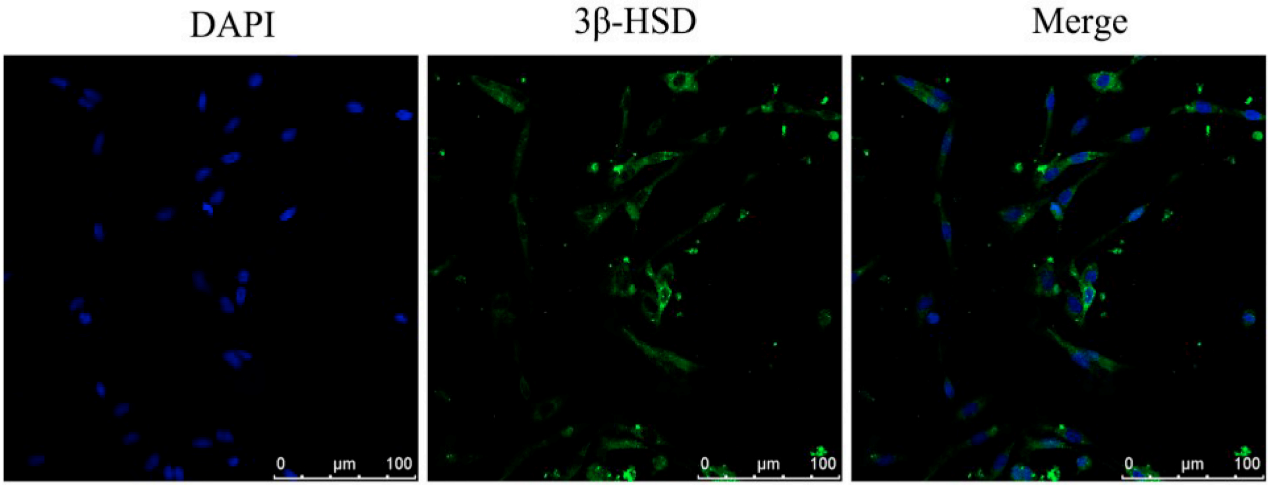

Supplementary Figure 2: Identification of primary cultured Leydig cells by immunofluorescence. Blue fluorescence represented nucleus of isolated cells stained by DAPI, green fluorescence indicated the Leydig cells by 3β-HSD.

Supplementary Table 1: Primer sequences used for PCR

Gene	Primers	Sequences
β-actin	Forward	5′ CCGGCATGTGCAAAGC 3′
	Reverse	5′ CACTGACCACCCTAACCTACC3′
NRF 1	Forward	5′ TATGGCGGAAGTAATGAAAGACG 3′
	Reverse	5′ CAACGTAAGCTCTGCCTTGTT 3′
StAR	Forward	5′ CAATTCTAACAACCGAAAG 3′
	Reverse	5′ TTGCTGCCTACCCTAA 3′

Supplementary Table 2: Primer sequences used for Chip-PCR

Gene	Primers	Sequences
cytc	Forward	5′ TATGGCGGAAGTAATGAAAGACG 3′
	Reverse	5′ CAACGTAAGCTCTGCCTTGTT 3′
StAR	Forward	5′ CAATTCTAACAACCGAAAG 3′
	Reverse	5′ TTGCTGCCTACCCTAA 3′
